# Ge_0.57_Ti_0.43_O_2_: a new high-pressure material with rutile-type crystal structure

**DOI:** 10.1107/S2056989018008988

**Published:** 2018-06-26

**Authors:** Emil Stoyanov, Kurt Leinenweber, Thomas L. Groy, Abds-Sami Malik

**Affiliations:** a Sandvik Hyperion, 6325 Huntley Road, Worthington, OH 43085, USA; b Eyring Materials Center, Arizona State University, Tempe, AZ 85287-1604, USA; c School of Molecular Sciences, PSD-102 MS-871604, Arizona State University, Tempe, AZ 85287-1604, USA

**Keywords:** titania-germania, solid solution, rutile structure, high pressure, high temperature, crystal structure

## Abstract

Single crystals of a GeO_2_–TiO_2_ solid solution with the corresponding composition Ge_0.57_Ti_0.43_O_2_ (germanium titanium tetra­oxide) were obtained by devitrification of germania-titania glass at high pressure and temperature. The new compound crystallizes in the rutile structure type (space group *P*4_2_/*mnm*), where Ge and Ti share the same position *M* (site symmetry *m.mm*), with occupancy values of 0.57 (3) and 0.43 (3), respectively, and one O-atom position (*m*.2*m*). The *M* site is in a sixfold O-atom coordination and, as in the original TiO_2_ rutile structure, an elongation of the O—*M*—O bonds along the *c*-axis direction of the coordination polyhedron and deviation of the angles from 90° lead to a decrease in the coordination symmetry from octa­hedral to tetra­gonal. The Ge and Ti atoms are fully disordered in the structure, which indicates that the rutile structure is surprisingly pliant given the differing sizes of the two cations.

## Abstract 2

Ge_0.57_Ti_0.43_O_2_ adopts the rutile structure type with Ge and Ti sites disordered on one position.

## Chemical context   

At ambient pressure, the GeO_2_–TiO_2_ phase diagram shows the formation of three phases: rutile-type GeO_2_, stable up to 1323 K, *β*-quartz-type GeO_2_, stable above 1323 K and TiO_2_ in the form of rutile. A metastable *α*-quartz-type structured GeO_2_ has also been reported as the result of the cooling of the *β*-quartz-type structure (Sarver, 1961 ▸). Additionally, at ambient pressure GeO_2_ and TiO_2_ exhibit only limited mutual solubility. The GeO_2_–TiO_2_ phase diagram at elevated pressures and temperatures has not been studied in great detail and the mutual solubility of Ge and Ti in the phases stable at these conditions is still largely unknown. GeO_2_ is dimorphous at ambient atmospheric conditions, represented by both rutile-type and *α*-quartz-structured phases depending on the temperature, but with increasing pressure the GeO_2_ rutile becomes more stable, and is the primary phase above two GPa (Micoulaut *et al.*, 2006 ▸). At pressures above 25 GPa, the tetra­gonal rutile-type phase transforms into an ortho­rhom­bic CaCl_2_-type phase (Haines *et al.*, 2000 ▸). TiO_2_ rutile undergoes two phase transitions under high pressure of up to 12 GPa: rutile-to-α-PbO_2_-type at around 7 GPa and α-PbO_2_-to-baddeleyite at 12 GPa (Gerward & Staun Olsen, 1997 ▸). We synthesized the title compound while investigating the GeO_2_–TiO_2_ phase diagram at a pressure of 8 GPa at 2028 K by means of the multi-anvil high-pressure technique. Instead of forming Ge-bearing TiO_2_ and Ti-bearing GeO_2_, we discovered that the high pressure and temperature conditions led to the formation of a crystalline, single solid-solution material. At temperatures above 1873 K, crystal growth was significant and high-quality single crystals of the solid solution with a composition near TiGeO_4_ could be obtained.

## Structural commentary   

**Figure 1:**
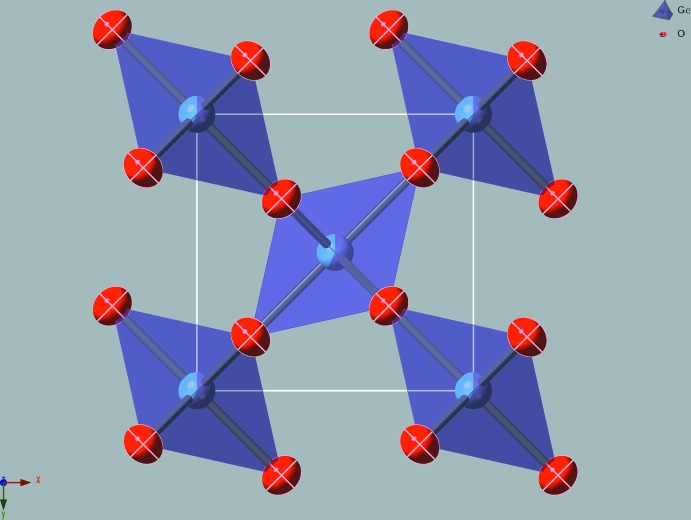
View of the structure of Ge_0.57_Ti_0.43_O_2_ looking down the *c* axis. The unit cell is outlined in white. The red ellipsoids represent the oxygen atoms and show the orientation of the displacement ellipsoids for 99% probability. The Ti atom is represented by light blue and the Ge atom by purple, with the percentage occupancy of the *M* site represented as a pie chart on the atom. *M*O_6_ octa­hedra are represented as transparent polyhedra.

**Figure 2:**
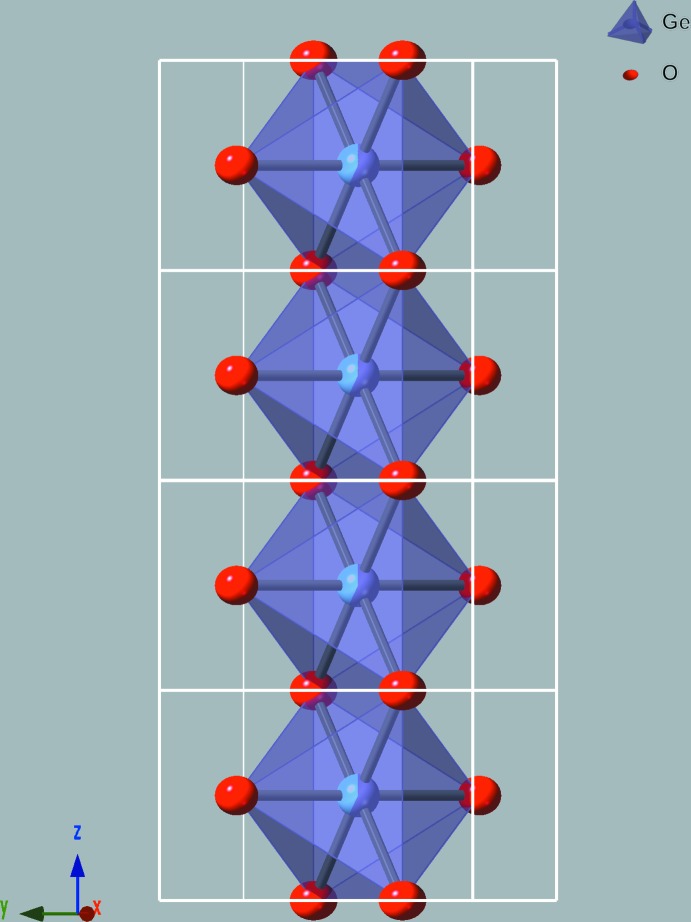
View of the edge-sharing chain of *M*O_6_ octa­hedra in Ge_0.57_Ti_0.43_O_2_. The symbols are the same as in Fig. 1 ▸.

The crystal structure of Ge_0.57_Ti_0.43_O_2_ corresponds to the TiO_2_ rutile type (space group *P*4_2_/*mnm*). The shared metal site *M* is in Wyckoff position *2a* and is surrounded by six O atoms, thus forming a sixfold coordination polyhedron. 57% of the *2a* positions are occupied by Ge and the remaining 43% are occupied by Ti. Each oxygen atom occupies a *4f* position and is surrounded by three *M* sites, forming triangular *M*O_3_ groups in the (110) lattice plane (Fig. 1 ▸). The structure is represented by chains of edge-sharing *M*O_6_ octa­hedra running parallel to the *c*-axis direction (Fig. 2 ▸) and connected to each other by shared corners. Relevant bond lengths and angles are presented in Table 1 ▸. In the *M*O_6_ coordination polyhedra, the *M*—O distances in the *xy* plane are 1.9080 (12) Å, while the *M*—O distances along the *z* axis increase to 1.9441 (19) Å. The *M*⋯*M* distances are equal to 2.9121 (13) Å. The elongation of O—*M*—O bonds along the *z* direction of the *M*O_6_ coordination polyhedron and the deviation of the angles from 90° lead to a decrease in point group symmetry from octa­hedral *O_h_* to tetra­gonal *D*_4*h*_. The unit-cell volume of Ge_0.57_Ti_0.43_O_2_ [58.79 (6) Å^3^] falls in between the rutile-type GeO_2_ [55.3424 (17) Å^3^] (Gullikson *et al.*, 2015 ▸) and TiO_2_ rutile [62.435 Å^3^] (Howard *et al.*, 1991 ▸) and indicates a linear relationship between the unit-cell volume and molar fraction of GeO_2_, adhering to Vegard’s Law.

The somewhat large difference in the ionic radii of the sixfold coordinated Ge^4+^ and Ti^4+^ (0.53 and 0.605 Å, respectively; Shannon, 1976 ▸) may be the reason for the limited mutual solubility of Ge and Ti in the rutile structured oxides at ambient pressure. This might explain why the single solid-solution phase is absent in the GeO_2_–TiO_2_ system, and why the synthesis of a material with composition near TiGeO_4_ requires high-pressure and high-temperature conditions. Disordering at high temperatures (significantly above the ambient-pressure melting point) could assist in the stability of the solid solution even with the two different sized cations.

## Synthesis and crystallization   

The title compound was synthesized by using an industrial multi-anvil high-pressure apparatus. The starting material was a GeO_2_–TiO_2_ glass produced from the corresponding oxide powders with a molar ratio of 60:40 (Sem-Com Company, Toledo, OH). A Pt foil capsule was loaded with the powdered glass and was subjected to high-pressure/high-temperature (HPHT) conditions of 8 GPa and 2028 K for 30 minutes, followed by cooling for 15 minutes to room temperature and releasing pressure non-isobarically to atmospheric pressure to recover the sample. The temperature was monitored with a W3%Re-W26%Re (C-type) thermocouple. The pressure was estimated by recovering and analyzing SiO_2_–GeO_2_ glass that was loaded in the Pt foil capsule and pressed in the same high-pressure cell. Thus, the pressure standard and the GeO_2_–TiO_2_ glass were treated at the same conditions. The details on the pressure calibration technique can be found elsewhere (Gullikson *et al.*, 2015 ▸; Leinenweber *et al.*, 2015 ▸). The applied temperature was sufficient to produce high-quality single crystals with uniform extinction in the optical microscope. A clear colourless tabular-like crystal from the recovered GeO_2_–TiO_2_ sample was used for the X-ray crystallographic analysis.

## Refinement   

Crystal data, data collection and structure refinement details are summarized in Table 2 ▸. Structure data were standardized according to the *STRUCTURE-TIDY* program (Gelato & Parthé, 1987 ▸).

## Supplementary Material

Crystal structure: contains datablock(s) global, I. DOI: 10.1107/S2056989018008988/wm5451sup1.cif

Structure factors: contains datablock(s) I. DOI: 10.1107/S2056989018008988/wm5451Isup2.hkl

CCDC reference: 1850471

Additional supporting information:  crystallographic information; 3D view; checkCIF report

## Figures and Tables

**Table 1:** Selected geometric parameters (Å, °)

Ge1—O1^i^	1.9080 (12)	Ge1—Ti1^ii^	2.9121 (13)
Ge1—O1	1.9441 (19)		
			
O1^i^—Ge1—O1^iii^	99.48 (8)	Ti1^ii^—Ge1—Ti1^vi^	180
O1^i^—Ge1—O1^iv^	80.52 (8)	Ti1^vii^—O1—Ti1^viii^	99.48 (8)
O1^v^—Ge1—O1	180	Ge1^vii^—O1—Ge1	130.26 (4)

Symmetry codes: (i) 
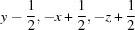
; (ii) 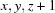; (iii) 
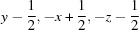
; (iv) 
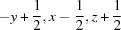
; (v) 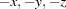; (vi) 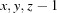; (vii) 
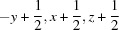
; (viii) 
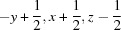
.

**Table 2:** Experimental details

Crystal data	
Chemical formula	Ge_1.14_Ti_0.86_O_4_
*M*_r_	187.88
Crystal system, space group	Tetragonal, *P*4_2_/*m* *n* *m*
Temperature (K)	298
*a*, *c* (Å)	4.493 (2), 2.9121 (13)
*V* (Å^3^)	58.79 (6)
*Z*	1
Radiation type	Mo *K*α
μ (mm^−1^)	17.23
Crystal size (mm)	0.08 × 0.08 × 0.07

Data collection	
Diffractometer	Bruker SMART APEX
Absorption correction	Multi-scan (*SADABS*; Bruker, 2014 ▸)
*T*_min_, *T*_max_	0.31, 0.40
No. of measured, independent and observed [*I* > 2σ(*I*)] reflections	722, 77, 76
*R*_int_	0.022
(sin θ/λ)_max_ (Å^−1^)	0.772

Refinement	
*R*[*F* ^2^ > 2σ(*F* ^2^)], *wR*(*F* ^2^), *S*	0.018, 0.050, 1.20
No. of reflections	77
No. of parameters	10
Δρ_max_, Δρ_min_ (e Å^−3^)	0.60, −0.93

Computer programs: *APEX2* and *SAINT* (Bruker, 2014 ▸), *SHELXT* (Sheldrick, 2015*a* ▸), *SHELXL2014* (Sheldrick, 2015*b* ▸), *CrystalMaker* (Palmer, 2015 ▸) and *publCIF* (Westrip, 2010 ▸).

## supplementary crystallographic information

### Crystal data

**Table:**

Ge_1.14_Ti_0.86_O_4_	*D*_x_ = 5.307 Mg m^−^^3^
*M**_r_* = 187.88	Mo *K*α radiation, λ = 0.71073 Å
Tetragonal, *P*4_2_/*m**n**m*	Cell parameters from 538 reflections
*a* = 4.493 (2) Å	θ = 4.5–33.3°
*c* = 2.9121 (13) Å	µ = 17.23 mm^−^^1^
*V* = 58.79 (6) Å^3^	*T* = 298 K
*Z* = 1	Tabular, clear colourless
*F*(000) = 87	0.08 × 0.08 × 0.07 mm

### Data collection

**Table:**

Bruker SMART APEX diffractometer	77 independent reflections
Radiation source: fine-focus sealed tube, sealed tube	76 reflections with *I* > 2σ(*I*)
Graphite monochromator	*R*_int_ = 0.022
Detector resolution: 8.3330 pixels mm^-1^	θ_max_ = 33.3°, θ_min_ = 6.4°
ω and φ scans	*h* = −6→6
Absorption correction: multi-scan (*SADABS*; Bruker, 2014)	*k* = −6→6
*T*_min_ = 0.31, *T*_max_ = 0.40	*l* = −4→4
722 measured reflections	

### Refinement

**Table:**

Refinement on *F*^2^	Primary atom site location: iterative
Least-squares matrix: full	Secondary atom site location: notdet
*R*[*F*^2^ > 2σ(*F*^2^)] = 0.018	*w* = 1/[σ^2^(*F*_o_^2^) + (0.0347*P*)^2^ + 0.0217*P*] where *P* = (*F*_o_^2^ + 2*F*_c_^2^)/3
*wR*(*F*^2^) = 0.050	(Δ/σ)_max_ < 0.001
*S* = 1.20	Δρ_max_ = 0.60 e Å^−^^3^
77 reflections	Δρ_min_ = −0.93 e Å^−^^3^
10 parameters	Extinction correction: SHELXL2014 (Sheldrick, 2015*b*)
0 restraints	Extinction coefficient: 0.58 (7)

### Special details

**Table:**

Geometry. All esds (except the esd in the dihedral angle between two l.s. planes) are estimated using the full covariance matrix. The cell esds are taken into account individually in the estimation of esds in distances, angles and torsion angles; correlations between esds in cell parameters are only used when they are defined by crystal symmetry. An approximate (isotropic) treatment of cell esds is used for estimating esds involving l.s. planes.

### Fractional atomic coordinates and isotropic or equivalent isotropic displacement parameters (Å^2^)

**Table:**

	*x*	*y*	*z*	*U*_iso_*/*U*_eq_	Occ. (<1)
Ge1	0	0	0	0.0049 (3)	0.57 (3)
Ti1	0	0	0	0.0049 (3)	0.43 (3)
O1	0.3059 (3)	0.3059 (3)	0	0.0077 (6)	

### Atomic displacement parameters (Å^2^)

**Table:**

	*U*^11^	*U*^22^	*U*^33^	*U*^12^	*U*^13^	*U*^23^
Ge1	0.0059 (4)	0.0059 (4)	0.0029 (4)	−0.00019 (9)	0	0
Ti1	0.0059 (4)	0.0059 (4)	0.0029 (4)	−0.00019 (9)	0	0
O1	0.0084 (7)	0.0084 (7)	0.0065 (8)	−0.0014 (5)	0	0

### Geometric parameters (Å, º)

**Table:**

Ge1—O1^i^	1.9080 (12)	Ge1—Ti1^vii^	2.9121 (13)
Ge1—O1^ii^	1.9080 (12)	Ge1—Ge1^vi^	2.9121 (13)
Ge1—O1^iii^	1.9080 (12)	Ge1—Ge1^vii^	2.9121 (13)
Ge1—O1^iv^	1.9080 (12)	O1—Ti1^viii^	1.9080 (12)
Ge1—O1^v^	1.9440 (19)	O1—Ge1^viii^	1.9080 (12)
Ge1—O1	1.9441 (19)	O1—Ti1^ix^	1.9080 (12)
Ge1—Ti1^vi^	2.9121 (13)	O1—Ge1^ix^	1.9080 (12)
			
O1^i^—Ge1—O1^ii^	99.48 (8)	O1^i^—Ge1—Ge1^vi^	40.26 (4)
O1^i^—Ge1—O1^iii^	80.52 (8)	O1^ii^—Ge1—Ge1^vi^	139.74 (4)
O1^ii^—Ge1—O1^iii^	180.0	O1^iii^—Ge1—Ge1^vi^	40.26 (4)
O1^i^—Ge1—O1^iv^	180.0	O1^iv^—Ge1—Ge1^vi^	139.74 (4)
O1^ii^—Ge1—O1^iv^	80.52 (8)	O1^v^—Ge1—Ge1^vi^	90.0
O1^iii^—Ge1—O1^iv^	99.48 (8)	O1—Ge1—Ge1^vi^	90.0
O1^i^—Ge1—O1^v^	90.0	Ti1^vi^—Ge1—Ge1^vi^	0
O1^ii^—Ge1—O1^v^	90.0	Ti1^vii^—Ge1—Ge1^vi^	180.0
O1^iii^—Ge1—O1^v^	90.0	O1^i^—Ge1—Ge1^vii^	139.74 (4)
O1^iv^—Ge1—O1^v^	90.0	O1^ii^—Ge1—Ge1^vii^	40.26 (4)
O1^i^—Ge1—O1	90.0	O1^iii^—Ge1—Ge1^vii^	139.74 (4)
O1^ii^—Ge1—O1	90.0	O1^iv^—Ge1—Ge1^vii^	40.26 (4)
O1^iii^—Ge1—O1	90.0	O1^v^—Ge1—Ge1^vii^	90.0
O1^iv^—Ge1—O1	90.0	O1—Ge1—Ge1^vii^	90.0
O1^v^—Ge1—O1	180.0	Ti1^vi^—Ge1—Ge1^vii^	180.0
O1^i^—Ge1—Ti1^vi^	40.26 (4)	Ti1^vii^—Ge1—Ge1^vii^	0
O1^ii^—Ge1—Ti1^vi^	139.74 (4)	Ge1^vi^—Ge1—Ge1^vii^	180.0
O1^iii^—Ge1—Ti1^vi^	40.26 (4)	Ti1^viii^—O1—Ge1^viii^	0
O1^iv^—Ge1—Ti1^vi^	139.74 (4)	Ti1^viii^—O1—Ti1^ix^	99.48 (8)
O1^v^—Ge1—Ti1^vi^	90.0	Ge1^viii^—O1—Ti1^ix^	99.48 (8)
O1—Ge1—Ti1^vi^	90.0	Ti1^viii^—O1—Ge1^ix^	99.5
O1^i^—Ge1—Ti1^vii^	139.74 (4)	Ge1^viii^—O1—Ge1^ix^	99.48 (8)
O1^ii^—Ge1—Ti1^vii^	40.26 (4)	Ti1^ix^—O1—Ge1^ix^	0
O1^iii^—Ge1—Ti1^vii^	139.74 (4)	Ti1^viii^—O1—Ge1	130.3
O1^iv^—Ge1—Ti1^vii^	40.26 (4)	Ge1^viii^—O1—Ge1	130.26 (4)
O1^v^—Ge1—Ti1^vii^	90.0	Ti1^ix^—O1—Ge1	130.3
O1—Ge1—Ti1^vii^	90.0	Ge1^ix^—O1—Ge1	130.26 (4)
Ti1^vi^—Ge1—Ti1^vii^	180.0		

Symmetry codes: (i) *y*−1/2, −*x*+1/2, −*z*+1/2; (ii) *y*−1/2, −*x*+1/2, −*z*−1/2; (iii) −*y*+1/2, *x*−1/2, *z*+1/2; (iv) −*y*+1/2, *x*−1/2, *z*−1/2; (v) −*x*, −*y*, −*z*; (vi) *x*, *y*, *z*+1; (vii) *x*, *y*, *z*−1; (viii) −*y*+1/2, *x*+1/2, *z*+1/2; (ix) −*y*+1/2, *x*+1/2, *z*−1/2.

### Atomic coordinates and equivalent isotropic atomic displacement parameters (Å^2^) for TiGeO_4_. *U*(eq) is defined as one third of the trace of the orthogonalized *U_ij_* tensor.

**Table:**

	*x*/*a*	*y*/*b*	*z*/*c*	*U*(eq)
Ge(1)	0.0	0.0	0.0	0.0049 (3)
Ti(1)	0.0	0.0	0.0	0.0049 (3)
O(1)	0.3059 (3)	0.3059 (3)	0.0	0.0077 (6)

### Anisotropic atomic displacement parameters (Å^2^) for TiGeO_4_.

**Table:**

	*U*_11_	*U*_22_	*U*_33_	*U*_23_	*U*_13_	U_12_
Ge(1)	0.0059 (4)	0.0059 (4)	0.0029 (4)	0	0	-0.00019 (9)
Ti(1)	0.0059 (4)	0.0059 (4)	0.0029 (4)	0	0	-0.00019 (9)
O(1)	0.0084 (7)	0.0084 (7)	0.0065 (8)	0	0	-0.0014 (5)

### Selected interatomic distances (Å) and angles for TiGeO_4_.

**Table:**

Ge(1)-O(1)	1.9080 (12)	Ge(1)-O(1)	1.9441 (19)
Ge1-Ti1	2.9121 (13)	O1-Ti1	1.9080 (12)
O1-Ge1	1.9080 (12)		
O(1)-Ge(1)-O(1)	99.48 (8)	O(1)-Ge(1)-O(1)	80.52 (8)
O(1)-Ge(1)-O(1)	180.0	O(1)-Ge(1)-O(1)	90.0
O(1)-Ge(1)-Ti(1)	139.74 (4)	O(1)-Ge(1)-Ti(1)	40.26 (4)
O(1)-Ge(1)-Ti(1)	90.0	Ti(1)-Ge(1)-Ti(1)	180.0
Ge1-O1-Ti1	99.48 (8)	Ti1-O1-Ge1	130.26 (4)
